# Oral health in nursing students at Kilimanjaro Christian Medical Centre teaching hospital in Moshi, Tanzania

**DOI:** 10.1186/s12903-015-0008-8

**Published:** 2015-02-20

**Authors:** Deogratias Stanslaus Rwakatema, Kanankira Nnko Ananduni, Victor William Katiti, Marycelina Msuya, Juliet Chugulu, Gibson Kapanda

**Affiliations:** Faculty of Medicine, Kilimanjaro Christian Medical University College of Makumira University, Arusha, Tanzania; Department of Dentistry, Kilimanjaro Christian Medical Centre, Moshi, Tanzania; Faculty of Nursing, Kilimanjaro Christian Medical University College of Makumira University, Arusha, Tanzania; Faculty of allied Health Sciences, Kilimanjaro Christian Medical University College of Makumira University, Arusha, Tanzania

**Keywords:** Oral hygiene, Caries status, Oral health knowledge, Oral health practices, Nursing students, Tanzania

## Abstract

**Background:**

This study aimed to determine the prevalence and severity of dental caries, oral hygiene levels and assessment of the oral health knowledge and practices of nursing students at Kilimanjaro Christian Medical Centre teaching hospital in Moshi, Tanzania.

**Methods:**

A cross-sectional survey was done on 217 student nurse population at Kilimanjaro Christian Medical Centre Teaching Hospital in Moshi, Tanzania in 2014. Ethical approval was obtained from the Kilimanjaro Christian Medical University College Ethical Committee. A questionnaire probing on socio-demographic characteristics, knowledge and practices on selected oral health issues was administered to the students. Students were also examined for oral hygiene and dental caries using Simplified Oral Hygiene Index (OHI-S) and WHO 1997 recommended method respectively.

**Results:**

There were 214 (98.6%) respondents aged between 18 and 53 years (mean age was 27.2 SD ± 7.35 years). About 72% of the respondents were in the young age group (below 31 years), 63.1% were pursuing Diploma in Nursing while the rest were pursuing Bachelor of Science in Nursing. Although oral health knowledge of the respondents was generally poor, more students pursuing Bachelor of Science in Nursing had significant adequate oral health knowledge than those who were pursuing Diploma in Nursing (p = 0.05). Population Oral Hygiene Index- Simplified was 0.41 meaning good oral hygiene in the current population. Overall, caries prevalence was 40.2%. The mean population DMFT was 1.34 (SD ± 2.44). The decay component was 0.53 (SD ± 1.29), whereas the missing component was 0.67 (SD ±1.34) and filled component was 0.14 (SD ± 0.69). Significantly more students in the older age group had more missing and filled teeth than their counterparts in the young age group (p ≤0.05).

**Conclusion:**

Majority of the students in this population had good oral hygiene and a very low DMFT. There was poor basic oral health knowledge and poor recall visit to dental personnel. Curriculum development in these school programmes should strengthen or encompass comprehensive oral health education components. This will empower nursing professional with basic oral health knowledge and promotive oral health behaviors and hence to disseminate to the clients.

## Background

Prevalence and severity of dental caries in Tanzanian adult communities is still lower compared to western countries [1,2]. However, sucrose containing diet snacking has been reported to be on the increase in East Africa [3]. The ongoing trade liberalization in Tanzania may lead to an increase on access to refined sugary foods. This could lead to an increase in the prevalence of dental caries. Overwhelming periodontal conditions have been reported among the communities in Tanzania [4-6]. Dental caries and chronic periodontal diseases are the commonest global diseases associated with behavioral and social factors [1]. Hence public oriented prevention actions for these diseases should be a main stay [7]. Nursing care personnel training is among the crucial human most benevolent educations. By nature of their role nurses are in constant contact with the patients and have upper hand in the community health welfare in their respective domicile. In that perspective a nurse should be empowered with basic oral health knowledge and promotive oral health behaviors. These qualities once transferred to or modeled through nursing care may enhance or commence lifetime optimal oral health behavior in the patients.

Documenting nurse’s oral health status, knowledge and practice levels on oral health is important to establish their potential contribution on simple basic promotive oral health services to the community they serve. Currently there are no retrievable studies on the nursing personnel oral health status, knowledge and preventive oral health practices in East Africa. The aim of this study was to assess oral hygiene, caries status, knowledge and preventive oral health practices in nursing students at Kilimanjaro Christian Medical Centre Teaching Hospital in Moshi, Tanzania.

## Methods

A cross sectional study was done on 217 students comprising of a population of student nurses at Kilimanjaro Christian Medical Centre Teaching Hospital in 2014. Of these, 135 were pursuing Diploma in Nursing at the School of Nursing and 79 Bachelor of Science in Nursing at the Faculty of Nursing of the Kilimanjaro Christian Medical University College. This teaching hospital is located in urban area of Moshi, Tanzania. A population of nursing students at Kilimanjaro Christian Medical Centre Teaching Hospital in 2014 was finite and small, whence the whole population was recruited for the study. Ethical clearance was sought from the Kilimanjaro Christian Medical University College Ethical Committee, research clearance No. 655.

A questionnaire probing on specific socio-demographic characteristics, knowledge and practices on selected oral health issues was administered to the nursing students’ population. There were two oral health domains assessed. These were students’ oral health knowledge and oral health preventive practices. Six questions in the questionnaire were used to probe for oral health knowledge and four questions for preventive oral health practices (Table 1). The questionnaire used had a mixture of open-ended questions and some were structured close-ended questions. The used questions were adopted and modified from the previous study [8] to suit the current study design and the nature of the current target population. A questionnaire was administered at the same time to all of the participants in the respective class rooms of the schools involved. Authors were available in those class rooms to clarify on any question arising in regard to the questionnaire.

**Table 1:** **Distribution of student nurses’ responses on common dental knowledge and practices (n = 214)**

**Questions**	**Response on knowledge and practice**			
	**Correct**		**Incorrect**	
	**No.**	**%**	**No.**	**%**
**Questions on oral health knowledge:**				
Do you know what causes easy bleeding of gums?	21	9.8	193	90.2
How can you prevent your gum from bleeding?	68	31.8	146	68.2
Do you know what causes dental decay?	94	43.9	120	56.1
How can you prevent your teeth from decaying?	72	33.6	142	66.4
Once caries has occurred, how can you timely manage it?	101	47.2	113	52.8
What is the content of a tooth paste which prevents tooth decay?	158	73.8	56	26.2
**Questions on oral health practices:**				
When did you last visit dental personnel?	31	14.5	183	85.5
How often do you brush your teeth?	175	81.8	39	18.2
How often do you use dental floss?	83	38.8	131	61.2
Do you use a tooth paste to brush your teeth?	211	98.6	3	1.4

For open-ended questions which gauged oral health knowledge, “Gingivitis” was considered a correct response for causes of easy bleeding of gums. “Proper tooth brushing” was considered correct response knowledge for the prevention of easy bleeding of gums. “Frequent sucrose-containing diet consumption” was considered a correct response for the causes of dental caries. “Restriction of sucrose-containing diet consumption to major meals was considered a correct knowledge response for the prevention of dental caries. Other answers without the inclusion of restriction of sugary foods to major meals were considered unsatisfactory for the causes and prevention of dental caries. For structured close-ended question which gauged oral health knowledge, “Restoration of a carious tooth to avoid pain and disease progression” was considered a correct alternative response against the “extraction to avoid pain”. This was for the question which asked “Once caries has occurred, how can you timely manage it?” “Fluoride” was considered a correct knowledge response against “Magnesium” or “Zinc” alternative responses for the question which asked “what is the content of a tooth paste which prevents dental caries?”

To gauge for basic preventive oral health practices, structured close-ended questions were used. A recall visit of “one or less than a year ago” to dental personnel was considered a good oral health practice against alternative responses of “2 years ago or less” and of “more than 2 years ago”. Tooth brushing with a frequency of “twice a day or more” was considered a correct alternative preventive oral health response against “once a day or less” response. Admitting to the “use of tooth paste” was considered a correct oral health practice response against “not using tooth paste” response. “Daily” or “occasionally” were independently considered as a correct preventive oral health response against “never” alternative response for a question which asked “How often do you use dental floss?”

Nursing students were examined for oral hygiene and dental caries using Simplified oral hygiene index (OHI-S) [9] and WHO 1997 recommended methods respectively [10]. Written informed consent was obtained from all of the examined students. An assistant entered data in a pre-prepared form. Clinical examinations were carried out under natural daylight in a classroom. This was achieved by having a student nurse sit on a chair next to a window. Ordinary dental probes and mirrors were used. One dental specialist carried out all the clinical examinations of the subjects. A total of 20 student nurses were re-examined and Kappa test was used to evaluate intra-examiner reproducibility for dental caries. Plaque examination and gingival bleeding tends to modify the local environment. Debris Index and Calculus Index which comprise the OHI-S therefore, could have not been reliably re-tested. Therefore reproducibility test for OHI-S was not analyzed.

### Data analysis

All the questions asked for oral health knowledge and practices were given equal weight. For each question asked, a correct answer was given a one score point per the question. Incorrect answer response was given a zero score point. Response for open-ended questions which involved either “Yes” “or No” response subjected the respondent to further provide the intended correct answer of the stem question if the given response was “Yes”. If that was not given, the “No” response was considered the respondent’s response. “Don’t know or don’t remember” alternative responses scored zero point in the data analysis. At the discretion of the authors a cut-off point for oral health knowledge domain was placed at 3 scores points. For those who scored 3 points and above were regarded to have good oral health knowledge in that domain. Those who scored below the 3 points were regarded to have poor oral health knowledge. Cut-off point for basic preventive oral health practice was placed at 2 scores points. Those who scored below 2 points were regarded as having poor oral health practice and for those who scored 2 points and above were regarded as having good oral health practices. Chi-square test was used to evaluate for any significant differences between socio-demographic characteristics and levels of knowledge and practices.

Data for OHI-S and DMFT were entered in a computer and analyzed using Microsoft Excel 2007 and SPSS version 18 programmes. T-test for independent samples was used to compare the means of OHI-S of the socio-demographic characteristics studied. Shapiro-Wilk tests were performed with each independent variable and the dependent variable of individual DMFT to determine normality. The results of the Shapiro-Wilk tests showed that the tested variables were not normally distributed (p-values < 0.05); therefore non-parametric test (Mann-Whitney U) was used to test the association of the DMFT with the socio-demographic characteristics studied. A p-value of equal or less than 0.05 was considered significant in this study.

## Results

Out of 217 student nurses population administered with questionnaires, 214 (98.6%) returned the questionnaire with complete answers. These were 25 (11.7%) male students and 189 (88.3%) female students. The mean age of the respondents was 27.2 (SD ± 7.35) years (range: 18-53 years). The specific socio-demographic characteristics of the respondents are presented in Table 2. In the respective domains respondents of thirty years and below constituted 72.4% of the respondents. This population had equal proportions (50%) of students having Ordinary or Advanced levels of Secondary School Education. Responses for questions probing on oral health knowledge and practice are presented in Table 1. Less than half of the respondents had incorrect responses on the causes of dental caries and chronic periodontal diseases. Only 14.5% of the respondents had correct responses in regard to recall visit to dental personnel. About 73% of the respondents responded correctly that fluoride is an important constituent of tooth paste that can prevents dental caries (Table 1). Likewise majority of the students answered correctly that they were brushing their teeth twice per day or more (Table 1). Almost all of the respondents answered correctly that they were using toothpastes during tooth brushing (Table 1). Less than half of the respondents 101 (47.2%) had adequate oral health knowledge. Those who were pursuing Bachelor of Science in Nursing had significantly adequate oral health knowledge than those who were pursuing Diploma in Nursing (Table 2). Majority of the respondents 165 (77.1%) had good oral health practice with no significant association across the socio-demographic characteristics studied.

**Table 2:** **Distribution of demographic characteristics of student nurses by levels of their oral health knowledge and practice (n = 214)**

**Socio-demographics**	**Oral health knowledge**				**Preventive oral health practice**				**Total number of respondents**	
	**Good**		**Poor**		**Good**		**Poor**			
	**No.**	**%**	**No.**	**%**	**No.**	**%**	**No.**	**%**	**No.**	**%**
Young (18-30 years)	69	44.5	86	55.5	118	76.1	37	23.9	155	72.4
Older (over 30 years	32	54.2	27	45.8	47	79.7	12	20.3	59	27.6
P value	0.26				0.71					
Ordinary secondary education	51	47.7	56	52.3	82	76.6	25	23.4	107	50.0
Advanced Secondary education	50	46.7	57	53.3	83	77.6	24	22.4	107	50.0
P value	0.89				0.87					
Diploma Nursing	55	40.7	80	59.3	99	73.3	36	26.7	135	63.1
BSc Nursing	46	58.7	33	41.8	66	83.5	13	16.5	79	36.9
P value	*0.01				0.09					
Population studied	101	47.2	113	52.8	165	77.1	49	22.9	214	100.0

Statistics: Chi-square test;* = p < 0.05.

A total of 214 nursing students who responded to the questionnaire were all examined for oral hygiene and caries status. The intra-examiner reproducibility test for caries examination produced a Kappa score of 0.9 indicating high reproducibility. Generally the groups mean Oral Hygiene Index (OHI-S) were very low with no significant difference across the socio-demographic characteristics analyzed (Table 3). The population Oral Hygiene Index (OHI-S) was 0.41 (Table 3). The overall caries prevalence in the population was 40.2% (Table 3). Students afflicted with caries were significantly more in those pursuing Diploma in Nursing than in the students pursuing Bachelor of Science in Nursing (Table 3). Descriptive statistics for DMFT in relation to socio-demographic characteristics studied are summarized in Table 4. Mann Whitney U test showed that significantly more students in the older age group had more missing and filled teeth than in the young age group while significantly more students undertaking Bachelor of Science in Nursing had higher DMFT than their counterparts in Diploma of Nursing School and students undertaking Bachelor of Science in Nursing had significantly more filled teeth than their counterpart in Diploma of Nursing School (Table 5). The overall mean DMFT of the population was 1.34 (SD ± 2.44) with a decay component of 0.53 (SD ± 1.29), a missing component of 0.67 (SD ± 1.34) and a filled component of 0.14 (SD ± 0.69).

**Table 3:** **Distribution of socio-demographic characteristics of student nurses (n = 214) by their Oral Hygiene Index (OHI-S) and caries experience**

**Socio-demographic characteristics**	**♦Mean OHI-S**	**♦♦Caries status**					
		**Caries positive**		**Caries free**		**Total**	
		**No**	**%**	**No**	**%**	**No**	**%**
Young (18-30 years)	0.38	58	67.4	28	32.6	86	40.2
Older (over 30 years	0.47	97	75.8	31	24.2	128	59.8
P value	0.26	0.18					
Ordinary secondary education	0.44	50	46.7	57	53.3	107	50.0
Advanced Secondary education	0.38	36	33.6	71	66.4	107	50.0
P value	0.39	* 0.05					
Diploma Nursing	0.37	49	36.3	86	63.7	135	63.1
BSc Nursing	0.47	37	46.8	42	53.2	79	36.9
P value	0.15	0.13					
Population	0.41	86	40.2	128	59.8	214	100

♦ = Statistics: Independent sample T-test.

♦♦ = Statistics: Chi-square test; * = p ≤ 0.05.

**Table 4:** **The group means decayed (D), missing (M), filled (F) teeth and DMFT by socio-demographic characteristics in 214 student nurses**

**Socio-demographic characteristics**	**Number**	**Decayed (D)**	**Missing (M)**	**Filled (F)**	**DMFT**
Young (18-30 yrs)	155	0.52	0.48	0.05	1.04
Older (31 yrs & above)	59	0.56	1.17	0.41	2.14
Ordinary secondary education	107	0.58	0.74	0.23	1.55
Advanced Secondary education	107	0.48	0.60	0.06	1.13
Diploma Nursing	135	0.44	0.48	0.04	0.96
BSc Nursing	79	0.67	0.99	0.33	1.99
Population studied	214	0.53	0.67	0.14	1.34

**Table 5:** **The group mean rank for decayed (D), missing (M), filled (F) teeth and DMFT by socio-demographic characteristics in 214 student nurses**

**Socio-demographic characteristics**	**Number**	**Mean Rank**			
		**Decayed (D)**	**Missing (M)**	**Filled (F)**	**DMFT**
Young (18-30 yrs)	155	107.42	102.74	103.89	103.46
Older (31 yrs & above)	59	107.07	120.01	116.99	118.12
P value		0.969	*0.023	*0.001	0.08
Ordinary secondary education	107	110.06	110.67	110.56	113.85
Advanced Secondary education	107	104.94	104.33	104.44	101.15
P value		0.403	0.352	0.092	0.09
Diploma Nursing	135	105.84	103.36	102.86	101.83
BSc Nursing	79	110.33	114.58	115.42	117.20
P value		0.48	0.112	*0.001	*0.048

Statistics: Mann Whitney U-test; * = p ≤ 0.05.

## Discussion

Majority of the students in this population had good oral hygiene and a low caries experience. However, poor basic oral health knowledge and poor recall visit to dental professionals were observed.

Majority of the respondents in this study were female. This is not surprising since in Tanzanian communities, nursing professional is mostly liked by the females. Less than half of the respondents answered correctly to the following questions in regards to the basic knowledge and prevention of common gum diseases: “Do you know what causes easy bleeding of gums?”, “How can you prevent your gum from bleeding?” These were surprising findings considering the levels of college students. It is likely that the primary oral health information acquired by these students before they joined the college was basically instructive rather than educative. Some of the students in this study were already in Primary school in 1988 at the time when the introduction of oral health self-care in Tanzanian Primary school education curricula was officially effected [11]. Although not researched on, it is common to find people associating easy bleeding of gums on brushing teeth with the lack of Vitamin C among the communities in Tanzania. Probably this had a root from the domestic science information on scurvy taught in Primary schools that bleeding of gum is caused by lack of Vitamin C. Furthermore, after the introduction of oral health self-care in Primary school education in Tanzania, the targeted pupils were and still are those in Standard 1 (after completion of Kindergarten level) up to Standard 3 [12]. Lack of sustaining self-oral health care education through the Standard Seven (last Standard before joining secondary education) pupils may lead the now adolescent pupils to retain very little, if at all any, oral health self-care knowledge. Some of these children could have turned into adulthood laden with informal oral health care information from their parents or caregivers. Our experience has noted that informal oral health beliefs in Tanzania and probably the East Africans include brushing teeth for the sake of whitening teeth and preventing mouth foul smell. Unfortunately none of these beliefs have been researched on before despite being common among the East Africans. Qualitative studies involving Focus Group Discussions in these populations may shade light on these beliefs to plan for educative intervention measures. However, children could easily retain behaviour of brushing teeth twice a day demonstrated in their early primary school years based on what they see in their parents or caregivers without the sound knowledge behind the reason for tooth brushing. Less than half of the respondents also answered correctly to the following questions in regards to the basic knowledge on the cause and prevention of dental decay: “Do you know what causes dental decay?”, “How can you prevent your teeth from decaying?” These again were surprising findings in students at the college level. There are beliefs among Tanzanian adults that caries is caused by worms. It is not uncommon in Tanzania to find traditional healers demonstrating presence of worms in the unfortunate dental decay sufferer’s mouth using cheating method. They do this cunningly based on the beliefs of these unfortunate individuals. These beliefs are informally passed on to the children by the parents or caregivers. Lack of continuous educative oral health care information to all standard levels in primary schools in Tanzania could lead these beliefs to continue into adulthood. This is because children at the age of Standard I-III cannot easily recall the given oral health information when they turn into adolescence or adulthood.

Students were asked: “Once tooth decay has occurred, how can you timely treat it?” Less than half of the students responded correctly to the timely treatment of dental caries. This finding implied that majority of the students in these school programmes could not seek treatment for a decayed tooth in the early stages. Probably they could wait until the tooth is painful enough to seek dental care. This has demonstrated what has been reported in Tanzanian adults that majority of the people seek dental care for pain relief [13].

Majority of the students answered correctly that Fluoride is the component of the toothpaste that prevents dental caries. Fluoridated toothpaste has been demonstrated to prevent dental caries [14,15]. Usefulness of fluoridated toothpaste to prevent dental caries is currently disseminated through mass media advertisements. These advertisements promote fluoridated toothpaste brands for commercial purposes. This could have raised knowledge of the respondents on the importance of fluoride in toothpaste. A well tailored oral health education component using mass media can bring rapid acquisition of the knowledge and practice to the entire population.

Results in this study showed poor oral health knowledge in students with Ordinary Secondary education and those with Advanced Secondary education with no significant difference between the two levels. This may imply that secondary school education activities in this population did not compound oral health knowledge at any school level that could have translated into long-term optimal preventive oral health knowledge. Indeed, the current Secondary schools syllabuses in Tanzania offer very little in terms of self-care oral health [16]. What is taught to these students is more of introduction to the subject rather than oral health preventive activities. On the other hand, students undertaking Bachelor of Science in Nursing had significantly better oral health knowledge than in those pursuing Diploma in Nursing (p = 0.05). Review of the curriculum in these two schools showed a component of self-study requirement about general body health and cleanness in Bachelor of Science in nursing programme. Although not systematically organized, Bachelor of Science in nursing students’ efforts in the area of general body health and cleanliness studies could have increased their knowledge on the basic oral health self-care compared to the students pursuing Diploma in Nursing where such a component is missing. Generally our study findings indicate the presence of poor preventive dental knowledge in the studied population. Our findings concurred with the previous study in Tanzania where poor oral health knowledge in adults has been reported [17]. Therefore, there is the need to incorporate comprehensive oral health care subjects coupled with preventive oral health care activities in the nursing school curricula.

Preventive oral health behavior was gauged by four questions as shown in Table 1. Majority of the students responded correctly on preventive oral health practice in half of the questions used to gauge this domain. This oral health domain was not significantly associated with any of the socio-demographic characteristics (p > 0.05) (Table 2). However, these results indicated a very low recall visit of at least “one to less than a year” as evidenced by majority of the students who gave inappropriate practice responses on the question which asked “When did you last visit dental personnel?” (Table 1). This scenario has also been reported recently among the secondary school students in Tanzania with the same or lower age group [18]. Majority of the adults in Tanzania have been reported to seek dental care for pain relief [13]. Some of the patients seek dental care when the carious lesion has completely destroyed the tooth crown where extraction becomes the only treatment alternative. This calls for ‘once a year or more’ traditional recall visit to dental personnel recommendable in Tanzania. This is for the reason of prevention, early detection and intervention of dental diseases. Indeed this was advocated in the Policy Guidelines for Oral Health Care in Tanzania [19]. Current global knowledge on the dental recall visit suggests that dental recalls should depend on individual needs based on risk-assessment [20]. However, the current study on dental recall visits was based on the Policy Guidelines for Oral Health Care in Tanzania [19]. Education on the promotive behavior towards regular dental recall visit is needed in the community of the current study taking into consideration the role model towards patients it play.

Easy bleeding of gums is a sign of chronic inflammation of the gums. Proper regular tooth brushing of at least twice a day or more keeps away the easy bleeding of gums. Good oral hygiene observed in the current study could be due to the good behavior on the frequency of tooth brushing reported by the majority of the respondents. This was however, in discrepancy to the incorrect responses given by the majority of the respondents on the causes and prevention of easy bleeding gums (Table 1). It is likely that oral health practices in these students were carried out blindly without sound basic oral health knowledge behind. Similar results were observed before in Tanzanian adolescents [5]. Informal oral health information handed over from parents or caregivers to the children in Tanzania was and probably remains instructive rather than educative. This could explain the nature of these findings especially for some places in Tanzania. In these areas there is lack of universities’ oral health education and research activities to the public. This is contrary to elsewhere in Dar es Salaam and its surroundings where such activities are the mainstay.

Almost all of the students reported to use tooth paste during tooth brushing. It is possible in the current population setup to argue that modernization in the school environment could have drawn majority of the students to use toothpaste. However, dental flossing which is also a modern culture in this part of the world was correctly practiced by few students in this study. Probably the commercial promotion of fluoridated toothpaste brands through mass media that it whitens teeth, causes teeth to become stronger and prevent foul smell could have contributed to the use of toothpaste in the overwhelming majority of the students. Indeed majority of the students in this study answered correctly that fluoride is the component of toothpaste that prevent dental caries.

The overall caries prevalence in the population of the current study was 40.2%. This was slightly higher compared to the recent findings of 31.9% prevalence of dental caries in Tanzanian adults of 18 years and above [21]. Students afflicted with caries were significantly more in those with Ordinary Level of secondary school education than in those with Advanced Level of secondary school education. Reason for this result is not clear according to the design of the current study if the age difference is put to consideration. Probably students with Ordinary Level of secondary school education had developed more habits of frequent access to sucrose containing diet compared to those with Advanced Level of secondary school education. Significantly more students in the older age group had more missing and filled teeth than in the young age group. This was not surprising since the caries experience has been reported to increase with age [22]. Furthermore, significantly more students undertaking Bachelor of Science in Nursing had higher DMFT than their counterparts in Diploma of Nursing School. Students in Diploma of Nursing School programme are relatively young compared to those in Bachelor of nursing school programme. Their caries experience was likely to be lower compared to those in the Bachelor of Nursing School considering the age factor in relation to caries experience. However, there was no statistical significant difference of the mean decayed component across the studied socio-demographic characteristics (p > 0.05). This could be due to low dental recall visit reported in this population. These students were studying and working in the vicinity of dental services establishment at this institution. They could have easily accessed dental care which could have changed the current reported missing (M) and filling (F) components of the mean DMFT. Furthermore, this could also relate to the previous reported behaviour of seeking dental care if there is pain among Tanzanians [13]. However, significantly more filled teeth were found in students undertaking Bachelor of Science in Nursing than in their counterparts studying Diploma of Nursing. This could probably be explained by the component of self-study requirement about general body health and cleanness found in syllabus of students undertaking Bachelor of Science in Nursing. This could have motivated them to seek timely dental care of filling their decayed teeth.

The overall mean DMFT of the population and its components (Table 4) were in agreement with the previously reported findings in Tanzanian adult population [23] and elsewhere in the East African population [24]. These findings were relatively very low compared to the reported scenario in Western countries and in Asia [2]. However, seen against the presence of inadequate oral health knowledge and poor dental revisit reported in this study, the decay component of the current DMFT in this population is likely to increase. This study was limited to a group of nursing students’ population at Kilimanjaro Christian Medical Centre Teaching Hospital. Whence, the information emanated from this study may not be extrapolated to other institutions in Tanzania or elsewhere in East Africa. However, due to the lack of similar studies like the present in the nursing student populations of the East Africans, the current efforts should be encouraged in other institutions of this region.

## Conclusions

It can be concluded that although most of the students in this population had good oral hygiene and lower DMFT, they lacked adequate basic oral health knowledge. The recommended dental recall visits according to Tanzanian oral health care were poor. Curriculum development in these school programmes should strengthen or encompass comprehensive oral health education components. This will empower nursing professional with basic oral health knowledge and promotive oral health behaviors and hence to disseminate to the clients.
